# Membranous nephropathy caused by dimercaptosuccinic acid in a patient with Wilson’s disease: a case report and literature review

**DOI:** 10.1186/s12882-023-03201-6

**Published:** 2023-05-26

**Authors:** Xiang Li, FengXin Hu, Gaosi Xu

**Affiliations:** 1 Department of Nephrology, The Second Affiliated Hospital of Nanchang University, No. 1, Minde Road, Donghu District, Nanchang, P.R. China; 2 Department of Obstetrics & Gynecology, The University of Hong Kong-Shenzhen Hospital, Shenzhen, China

**Keywords:** Membranous nephropathy, Dimercaptosuccinic acid, Wilson’s disease

## Abstract

**Background:**

Dimercaptosuccinic acid (DMSA) therapy is a kind of chelation therapy for patients with Wilson ‘s disease (WD). While there have been reports of side effects associated with DMSA, the development of membranous nephropathy as a result of this therapy is uncommon.

**Case presentation:**

We present a case of a 19-year-old male patient with Wilson’s disease who experienced proteinuria while receiving long-term DMSA treatment. Further evaluation revealed abnormally low levels of serum ceruloplasmin and serum albumin, as well as a 24-hour urinary protein excretion of 4599.98 mg/24 h. A renal biopsy confirmed the presence of membranous nephropathy. After ruling out other potential causes, we determined that the patient’s membranous nephropathy was likely caused by DMSA. Following treatment with glucocorticoids, there was a significant reduction in proteinuria.

**Conclusion:**

This case highlights the possibility of DMSA-induced membranous nephropathy and the importance of considering this diagnosis in patients receiving DMSA treatment. Given the widespread use of DMSA in the treatment of Wilson’s disease, further research is needed to fully understand the potential role of this drug in the development of membranous nephropathy.

## Introduction

Dimercaptosuccinic acid (DMSA) is usually used to promote copper excretion in WD patients. Compared with other drugs for the treatment of WD, dimercaptosuccinic acid has mild adverse reactions, and it is rare to be forced to discontinue due to adverse reactions. At present, the main side effects of dimercaptosuccinic acid are as follows: (i) Neurological deterioration: mainly manifested as increased muscular tension, mental symptoms appear or worsen. (ii)digestive tract reactions: mainly manifested as fatigue, abdominal distension, and decreased appetite. (iii) Allergic reaction: mainly manifested as fever, drug rash. (iv) Bleeding: mainly manifested as gum, epistaxis, skin petechiae, and ecchymosis [1]. However, there is limited report on the potential renal side effects of DMSA. This report describes a patient with Wilson’s disease who was receiving long-term DMSA treatment and developed proteinuria, which was later confirmed through renal biopsy to be membranous nephropathy. The patient was successfully treated with glucocorticoids.

## Case report

A 19-year-old male with Wilson ‘s disease (WD) for 7 years was referred to our hospital with complaints of a 1-year history of foam urine. He was found to have proteinuria in a checkup 1 month ago. He was diagnosed with WD 7 years ago due to the appearance of corneal K-F ring and the reduction of blood ceruloplasmin. After that, the patient was regularly treated with DMSA for copper excretion. He denied a family history of genetic disease and D-penicillamine medication history. Physical examination revealed no significant abnormalities. Blood examinations showed normal blood cells. normal levels of serum creatinine (93.28µmol/L), blood glucose (5.23 µmol/L), triglyceride(2.29mmol/L), and total cholesterol (5.71 mmol/L), low levels of total protein (6.2 g/dl) and serum albumin (2.7 g/dl). Urinalysis showed that urine protein (2+), and 24-hour urinary protein was 4599.98 mg/24 h. Anti-nuclear antibody (ANA), antineutrophil cytoplasmic antibody (ANCA), and Anti phospholipase A2 receptor antibody(PLA2R) were negative. Tumor and infection indicators are normal. Doppler sonography showed no abnormality.

**Fig. 1:**
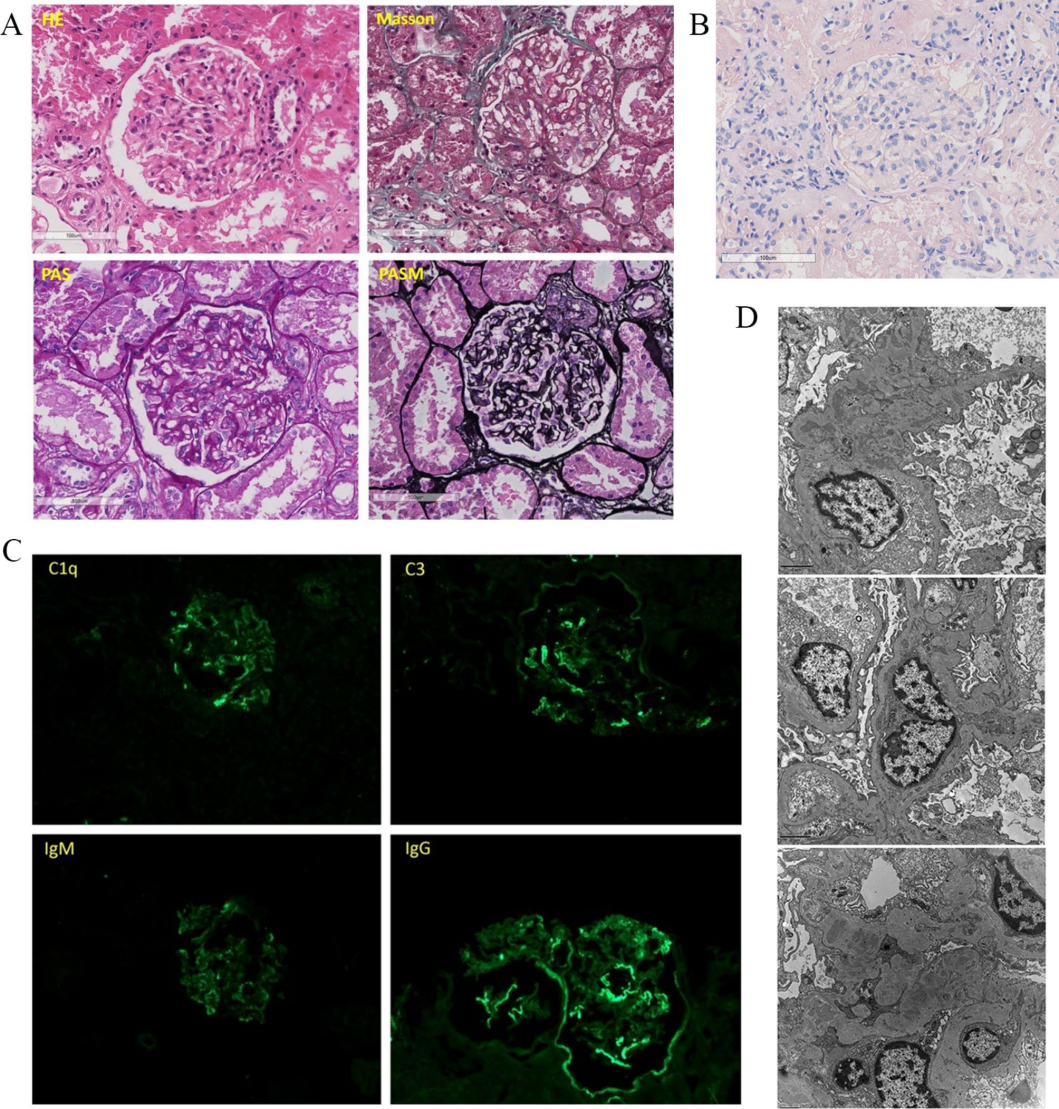
Histological images from the renal biopsy. (**A**) Staining of HE, Masson, PAS, and PASM. Light microscopy shows the glomerulus with thickening of basement membranes and spikes. (**B**) Staining of copper. No obvious copper deposition was found in renal tubular epithelial cells and glomerular sacs (**C**) Immunofluorescence micrographs. immunofluorescence microscopy shows C1q(++), C3(++), IgM(+), and IgG(++) deposited along the glomerular capillary. (**D**) Electron micrographs. Electron microscopy shows glomerular basement membrane irregularly thickened and podocyte foot process effacement. Electron-dense deposits were noted in the subepithelial, basement membrane, subendothelial and mesangial regions

**Fig. 2:**
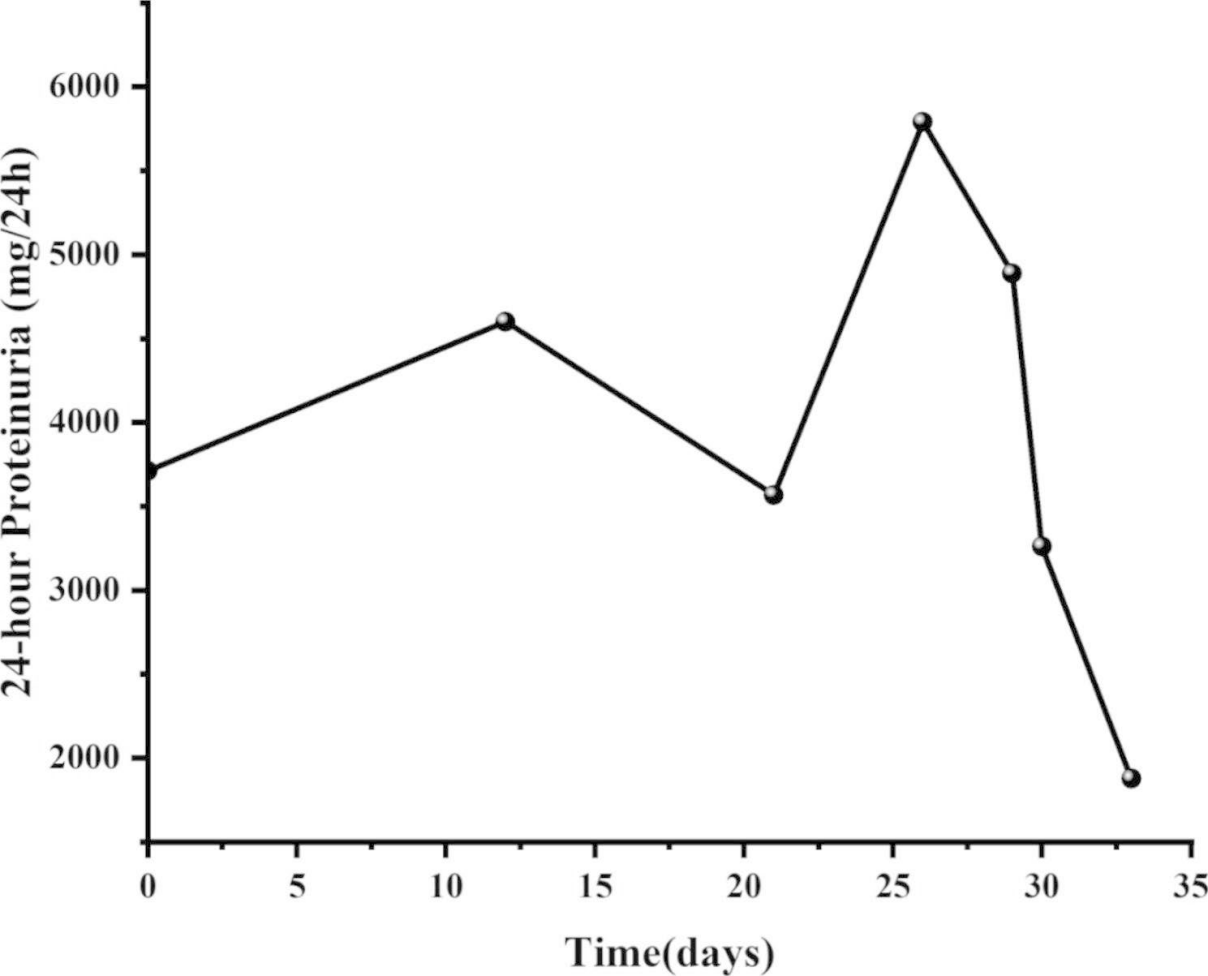
Efficacy evaluation, changes of the 24-hour proteinuria before and after treatments. Treatment of glucocorticoid initiated on the 28th day following admission to our hospital. We observed a significant reduction in 24-hour urinary protein excretion following treatment with glucocorticoids

A renal biopsy was performed. Light microscopy showed thickened glomerular basement membrane (GBM) and discrete “spike” formation. The capillary loops were open. And mesangial expansion with mesangial hypercellularity were observed (Fig. 1A). Immunofluorescence microscopy revealed granular deposits of IgG(2+), IgM(+), C1q(2+), and C3(2+) along the glomerular capillary wall. Protein absorption droplets can be seen in renal tubular epithelial cells (Fig. 1B). Furthermore, the test of copper staining was negative (Fig. 1C). Electron microscopy revealed glomerular basement membrane irregularly thickened and podocyte foot process effacement. Electron-dense deposits were noted in the subepithelial, basement membrane and mesangial regions (Fig. 1D). He was finally diagnosed with secondary MN caused by DMSA. Glucocorticoid (triamcinolone 32 mg/d) was initiated. The change of 24-hour urinary protein was recorded, and 24-hour urinary protein was significantly reduced after the treatment initiated on the 28th day following admission to our hospital (Fig. 2).

## Discussion

DMSA is a broad-spectrum metal chelating agent for the treatment of WD. In this case, after long-term use of DMSA, urinalysis showed proteinuria (2+) and 24-h urine protein was 4599.98 mg/24 h. Renal biopsy confirmed the diagnosis of membranous nephropathy was identified, and the laboratory tests demonstrated ANA (-), ANCA (-), and PLA2R (-). Tumor and infection indicators are normal. So, we ruled out the possibility of tumor and infection in the patient based on their clinical manifestations, tumor markers, infection indicators, blood tests, urine tests, and ultrasound results. At present, the patient is treated with oral medication to expel copper regularly, the blood copper is maintained at a low level, and the corneal K-F ring disappears. Copper staining of renal tissue showed no obvious copper particles were deposited in renal tubular epithelial cells and glomerular sacs. MN caused by WD was excluded, and there was no history of kidney disease or other renal involvement in patients. Therefore, we concluded that DMSA was the cause of membranous nephropathy in this patient.

Wilson’s disease (WD) is an inherited disorder that causes excessive accumulation of copper in the liver, brain, and other organs. The accumulation of toxic amounts of copper in the liver, brain, and other organs may cause various clinical conditions, often with prominent neurological, psychiatric, and liver-related symptoms [2]. Currently, the main treatment options for Wilson’s disease include D-penicillamine, trientine, zinc, and DMSA [3, 4] (Table 1). D-penicillamine is effective at removing copper from the body and can be used to treat all patients with symptoms of Wilson’s disease. However, it can cause a range of adverse reactions, including neurological deterioration in 10–20% of patients during the initial treatment phase. Early sensitivity reactions, such as fever, skin eruptions, lymph node swelling, neutropenia or thrombocytopenia, and protein in the urine, may occur during the first 1–3 weeks of treatment. Later effects of treatment with D-penicillamine may include nephrotoxicity, bone marrow toxicity, and dermatological toxicities. In 1969, trientine (triethylene tetramine dihydrochloride or 2,2,2-tetramine) was introduced as an alternative to D-penicillamine. Trientine is a chelator with a polyamine-like structure chemically distinct from D-penicillamine. It is similar to D-penicillamine in that it promotes the excretion of copper in the kidneys. Trientine is generally associated with fewer side effects than D-penicillamine, but it can still cause bone marrow depression [5], hepatotoxicity [6], and overly aggressive removal of copper, which can lead to neurological dysfunction. Besides, the high cost and lack of availability of trientine limit its use in China. Zinc works by inhibiting copper absorption in the intestine, It can be used as first-line treatment for asymptomatic patients, as well as maintenance treatment for ordinary patients and alternative treatment for penicillamine intolerant patients. Zinc is also effective in treating neurological symptoms associated with Wilson’s disease. It has few side effects, the most common of which are gastrointestinal irritation and numbness of the lips and limbs. Other potential adverse effects include decreased immune function and increased serum cholesterol and low density lipoprotein levels. In addition, zinc’s effects may be slow to manifest.

**Table 1:** Currently available oral treatments for Wilson’s disease

Drug	Mode of action	Side effects	
**D-Penicillamine**	General chelator induces renal excretion of copper	• Neurological deterioration • Fever and cutaneous eruptions, lymphadenopathy, neutropenia or thrombocytopenia, and proteinuria • Nephrotoxicity • Bone marrow toxicity • Dermatological toxicities	
**Trientine**	General chelator induces renal excretion of copper	• Bone marrow depression • Hepatotoxicity • Neurological dysfunction	
**Zinc**	Metallothionein inducer, blocks intestinal copper absorption	• Gastrointestinal irritation • Numbness of the lips and limbs • Decreased immune function • Increased serum cholesterol and low-density lipoprotein levels	
**Dimercaptosuccinic acid**	General chelator induces renal excretion of copper	• Neurological deterioration: mainly manifested as increased muscular tension, mental symptoms appear or worsen. • Digestive tract reactions: mainly manifested as fatigue, abdominal distension, and decreased appetite • Allergic reaction: mainly manifested as fever, drug rash. •Gum bleeding:	

Dimercaptosuccinic acid (succimer; DMSA), a water-soluble analog of dimercaprol, has been used since the 1950s as an antidote for heavy metal toxicity [7]. DMSA can form complexes with copper ions and oral DMSA significantly increased urinary copper excretion [8]. DMSA was first used as a copper chelator for WD in China [9] and there are substantial experiences with the use of DMSA for WD treatment in China [10]. At present, the known side effects include: (i) Neurological deterioration: mainly manifested as increased muscular tension, mental symptoms appear or worsen. (ii)digestive tract reactions: mainly manifested as fatigue, abdominal distension, and decreased appetite. (iii) Allergic reaction: mainly manifested as fever, drug rash. (iv) Bleeding: mainly manifested as gum, epistaxis, skin petechiae, and ecchymosis [1]. However, there are few reports about membranous nephropathy caused by DMSA. The mechanism of it leading to membranous nephropathy is unclear. So far, several medications have been known to cause membranous nephropathy, mainly including: gold therapy; Penicillamine and Bucillamine; Mercury; Captopril; and NSAIDs. Wherein the penicillamine and busilamine are known to cause MN. The mechanism by which penicillamine and bucillamine produce MN is unknown but may involve modification of the immune response and/or hapten formation. Captopril is an angiotensin-converting enzyme inhibitor (ACE-I) that is commonly used to treat hypertension and reduce proteinuria. It was reported that ACEI can induce MN attributed to a sulfhydryl group, which is unique to captopril among the ACE-Is but a feature that it shares with penicillamine and bucillamine [11]. Therefore, it is speculated that mechanism by which the drugs stimulate the response may involved in the thiol group of the drugs, which permits covalent bonding to cellular macromolecules [12]. The DMSA contains two active sulfhydryl groups and has strong affinity with metal ions. Mercapto groups can covalently bind to macromolecules, making drugs containing mercapto groups may be used as haptens to induce antibody production. However, the role of DMSA in secondary MN needs to be further studied, as the literature regarding this topic is limited.

Membranous nephropathy (MN) is a glomerular disorder typified by the accumulation of an abundance of immune complexes on the epithelial aspect of the glomerular capillary loop. The prevailing clinical presentation is that of nephrotic syndrome (NS). The incidence of secondary MN constitutes approximately 30% of all cases of MN [13]. In a review of nine published series on MN, 6.6% of patients presented drug-induced disease [14]. In a cohort, a drug-induced etiology was identified in 14% of patients [15]. Pathologic findings frequently pose challenges in discriminating primary MN from drug-induced MN (DIMN), thus underlining the pivotal role of obtaining comprehensive clinical details concerning medication utilization. The treatment of MN (DIMN) begins with discontinuation of the culprit drug. In some instances, such as with gold salts, penicillamine, and bucillamine, regular monitoring of urine is necessary due to the high incidence of proteinuria and MN. Immunosuppressive therapy may be necessary in cases of severe nephrotic syndrome (NS) symptoms or lack of improvement [11]. Although DMSA induced membranous nephropathy is rarely reported, the therapeutic approaches are akin to those utilized for most drug-induced membranous nephropathy cases. In our case, hormone therapy has been verified as efficacious.

## Conclusion

We report a rare case of membranous nephropathy caused by dimercaptosuccinic acid in a patient with Wilson’ s disease. DMSA are now widely used in the treatment of WD and can lead to side effects on kidneys, which rare reported and may go unrecognized. The diagnosis of DMSA-induced secondary MN is challenging and requires a proper laboratory work-up and histological testing. The further studies are needed to evaluate and comprehend the role of DMSA in this condition. In addition, we should use drugs cautiously according to drug safety and patient risk factors, and make timely judgments and active treatment when side effects occur. The case will provide a reference for side effects caused by DMSA.

## Data Availability

The datasets used and analyzed during the current study are available from the corresponding authors on reasonable request.

## Abbreviations

ACE-I: Angiotensin-converting enzyme inhibitor
DMSA: Dimercaptosuccinic acid
MN: Membranous nephropathy
WD: Wilson’s disease
